# The costs of monitoring trachoma elimination: Impact, surveillance, and trachomatous trichiasis (TT)-only surveys

**DOI:** 10.1371/journal.pntd.0007605

**Published:** 2019-09-05

**Authors:** Rachel D. Stelmach, Rebecca M. Flueckiger, John Shutt, Margaret Davide-Smith, Anthony W. Solomon, Lisa Rotondo, Aryc W. Mosher, Margaret Baker, Rebecca Willis, Jeremiah Ngondi

**Affiliations:** 1 Global Health Division, International Development Group, RTI International, Washington, District of Colombia, United States of America; 2 Global Health Division, International Development Group, RTI International, Atlanta, Georgia, United States of America; 3 Global Health Division, International Development Group, RTI International, Research Triangle Park, North Carolina, United States of America; 4 Clinical Research Department, London School of Hygiene & Tropical Medicine, London, United Kingdom; 5 Department of Control of Neglected Tropical Diseases, World Health Organization, Geneva, Switzerland; 6 Bureau for Global Health, United States Agency for International Development, Washington, District of Columbia, United States of America; 7 Task Force for Global Health, Atlanta, Georgia, United States of America; 8 Global Health Division, International Development Group, RTI International, Cambridge, United Kingdom; Mohammed Bin Rashid University of Medicine and Health Sciences, UNITED ARAB EMIRATES

## Abstract

**Background:**

Although trachoma causes more cases of preventable blindness than any other infectious disease, a combination of strategies is reducing its global prevalence. As a district moves toward eliminating trachoma as a public health problem, national programs conduct trachoma impact surveys (TIS) to assess whether to stop preventative interventions and trachoma surveillance surveys (TSS) to determine whether the prevalence of active trachoma has rebounded after interventions have halted. In some contexts, programs also conduct trachomatous trichiasis (TT)-only surveys. A few costing studies of trachoma prevalence surveys exist, but none examine TIS, TSS, or TT-only surveys.

**Methodology/Principal findings:**

We assessed the incremental financial cost to the national program of TIS, TSS, and TT-only surveys, which are standardized cluster-sampled prevalence surveys. We conducted a retrospective review of expenditures and grant disbursements for TIS and TSS in 322 evaluation units in 11 countries between 2011 and 2018. We also assessed the costs of three pilot and five standard TT-only surveys in four countries between 2017 and 2018. The median cost of TIS and TSS was $8,298 per evaluation unit [interquartile range (IQR): $6,532–$10,111, 2017 USD]. Based on a linear regression with bootstrapped confidence intervals, after controlling for country, costs per survey did not change significantly over time but did decline by $83 per survey implemented in a single round (95% CI: -$108 –-$63). Of total costs, 80% went to survey fieldwork; of that, 58% went towards per diems and 38% towards travel. TT-only surveys cost a median of $9,707 (IQR: $8,537–$11,635); within a given country, they cost slightly more (106% [IQR: 94%–136%]) than TIS and TSS.

**Conclusions/Significance:**

The World Health Organization requires trachoma prevalence estimates for validating the elimination of trachoma as a public health problem. This study will help programs improve their planning as they assemble resources for that effort.

## Author summary

The global health community is working to eliminate trachoma, a bacterial disease that causes blindness. The elimination strategy includes offering antibiotics to everyone living in communities with a high prevalence of active trachoma, also known as mass drug administration, and providing surgeries for people with advanced trachoma. The World Health Organization requires that countries conduct surveys to see whether a district has reached the prevalence benchmarks for stopping population-level prevention interventions against trachoma. After stopping these interventions, programs conduct more surveys to make sure that trachoma has remained at bay. Trachoma programs must include the costs of these surveys in their budgets. Although some estimates of the costs of baseline trachoma prevalence surveys already exist, none examine the costs of additional surveys required later in the program. In this paper, we analyze the costs of over 300 of these surveys. We find that they cost about $8,000-$10,000 per survey, varying widely by country. Variations are mostly driven by how much it costs to pay surveyors and transport them to survey sites. Costs did not appear to change over time. With the data that we present in this paper, national programs will be more prepared to plan for monitoring the elimination of trachoma.

## Introduction

Trachoma, caused by the bacterium *Chlamydia trachomatis*, leads to more cases of preventable blindness than any other infectious disease [1]. Through Surgery for trachomatous trichiasis (TT), Antibiotic treatment, Facial cleanliness, and Environmental improvement (the SAFE strategy), national programs are reducing the prevalence of trachoma in many countries worldwide. As of December 2018, the World Health Organization (WHO) had validated the elimination of trachoma as a public health problem in eight countries: Cambodia, Ghana, the Islamic Republic of Iran, Lao People’s Democratic Republic, Mexico, Morocco, Nepal, and Oman [2]. To eliminate trachoma as a public health problem, a country must meet three requirements: (1) a trachomatous inflammation—follicular (TF) prevalence sustained below 5% for at least two years in 1–9-year-olds without antibiotic mass drug administration in each formerly-endemic evaluation unit (EU), (2) a TT prevalence of less than 0.2% in people 15 years of age or older in each formerly-endemic EU, and (3) a system in place for identifying and treating new cases of TT [3]. In this definition, an EU usually contains 100,000–250,000 people.

Before validating elimination, WHO requires evidence that the prevalence of TF and TT very likely falls below the elimination thresholds. To generate this evidence, national programs often use surveys. Trachoma baseline surveys assess the prevalence of TF and TT before any intervention so that countries can decide which aspects of the SAFE strategy to implement, if any [4]. After national programs implement these interventions, they can conduct trachoma impact surveys (TIS) to assess TF and TT prevalence to see if a district can stop implementing the SAFE strategy at a population level. At least 24 months after stopping interventions, trachoma surveillance surveys (TSS) are conducted to ensure that trachoma has not re-emerged as a public health problem [5]. These surveys all use population-proportional-to-size cluster sampling methods from very similar standard methodologies [6]. In addition to these surveys, a TT-only survey methodology has been developed for use in certain epidemiological contexts where programs wish to assess the prevalence of TT separately from assessing the prevalence of TF [7]. National programs must factor the costs of all surveys into their annual and long-term budgets.

A few costing studies on trachoma prevalence surveys exist. One study examined the costs of a mix of baseline and impact surveys in eight countries in Africa [8], and another examined the costs of baseline surveys conducted by the Global Trachoma Mapping Project [9]. Neither of these, however, specifically examined TIS, TSS, or TT-only surveys. One (to-date-non-peer-reviewed) analysis of TIS costs does exist, but it only examines costs within the Amhara region of Ethiopia [10]. To add to the body of cost data available to national programs as their countries progress toward elimination, this study examines the costs of TIS and TSS in 11 countries and of TT-only surveys in four countries. To assist national programs with applying the findings to their unique contexts, the study further analyzes the cost components of these surveys and tests several hypotheses regarding factors that might drive differences in survey costs, such as country, calendar year, and years of implementation within a country. With these data, national programs will be better prepared to budget for the elimination of trachoma as a public health problem.

## Methods

### Data sources

This study assesses the incremental financial costs to the national program of TSS and TIS in 11 countries from 2012 to 2018. We examined all available surveys supported by partners on the United States Agency for International Development (USAID) projects for which we had access to cost data. We also assessed the incremental financial costs to the national program of three pilot TT-only surveys conducted in Cameroon, Tanzania, and Uganda and five standard TT-only surveys conducted in Nepal. All surveys were standardized—including the use of electronic data capture—through the Tropical Data system, which was developed as an evolution of the Global Trachoma Mapping Project.

### Data collection

The methods used in this paper draw on those of previous investigators, particularly those of Chen et al. [8]. We developed and piloted a standardized data extraction form in Excel that requested information on survey type, EU characteristics, start and end dates, and costs by line item. An EU represents the level at which a survey is powered to show results; in most settings, it corresponds with an implementation unit such as a district. We gathered costs using the ingredients approach [11]: each line item was associated with a unit count and a unit cost, which were multiplied to calculate the cost per line item. In addition, each line item was tagged with exactly one activity and one category, shown in Table 1. Since we assessed only the incremental cost of the surveys—that is, only additional costs directly related to undertaking the surveys beyond the usual costs of the national program—we excluded any costs that would have been incurred had the survey not taken place. This restriction excluded salaries and other remuneration of staff time at both central and field offices except for any per diems received by a staff member for survey-related activities; it also excluded the development and maintenance of the Tropical Data system. As we collected only financial costs to the program, we did not include economic costs or costs to society, such as the value of subjects’ time.

**Table 1:** Line item categories and activities.

	Name	Explanations and examples
**Activity**	Pre-meetings	Sensitizing communities or schools, planning surveys
	Training	Training of local survey teams and supervisors
	Survey fieldwork	Collecting data, supervision
	Post-meetings	Meeting to inform districts of results
	Survey design, data analysis, and report writing	Designing surveys, analyzing data, and report writing
	Other	Any other survey-related activity
**Category**	Supplies	Stationery, field equipment, tetracycline eye ointment, etc.
	Per diem	Per diems or honoraria, including lodging costs
	Travel	Vehicle rentals, fuel, etc.
	Other	Any other category of spending

This table shows the categories and activities in the data extraction form. Each line item was tagged with exactly one activity and one category.

Each national program often conducted multiple surveys of a given type in a single year. As all costs for a given survey type in each fiscal year were tracked as a block, we could not gather unique costs by EU. We therefore calculated the cost per survey for each line item by dividing its cost by the reported number of EUs for the country, fiscal year, and survey type [9]. Throughout this paper, a “survey” refers to one survey of a given type (TIS, TSS, or TT-only) implemented in an EU. (A fiscal year stretches from the October of the previous numeric calendar year through the September of the current calendar year; for example, fiscal year 2017 includes dates from October 1, 2016 through September 30, 2017. Since we gathered data on the actual calendar dates of the survey, we report years below in terms of calendar years for ease of interpretation.)

Local finance teams from each national program completed the data extraction form. Costs were recorded in the currency used in the national team’s records, either United States dollars (USD) or the local currency. Wherever possible, country teams provided actual expenditure data. Where surveys are funded through fixed obligation grants (FOGs), in which programs receive a pre-negotiated amount of money upon the completion of pre-arranged milestones, national programs often did not track their actual expenditures with the granularity we required for our analysis. Therefore, for surveys funded through FOGs, we used the line item amounts agreed upon in the FOG budgets, as those amounts reflect the amount disbursed for the surveys.

### Data analysis

A single researcher (RDS) reviewed the forms for completeness and consistency. Where questions arose, the researcher contacted the national programs for clarification. Data were cleaned and analyzed with R version 3.5.1. All costs were converted to USD using mean exchange rates over the period of survey activity as reported by XE.com [12] and then inflated or deflated to 2017 USD using the implicit gross domestic product price deflator [13]. Due to the often-skewed nature of costing data, we used the non-parametric Mann-Whitney test to evaluate the difference in costs between survey types and funding models. We also used linear regression to evaluate whether the number of years a country had been implementing TIS or TSS, the calendar year of implementation, or the number of surveys conducted at once affected the cost per survey. We bootstrapped the confidence intervals in these regressions with 100,000 replications using the bias-corrected and accelerated method [14,15]. This method provides a better estimate of confidence intervals on skewed data than other approaches, such as the simple percentile method [16].

For TT-only surveys pilot in Cameroon, Tanzania, and Uganda, we made two adjustments to the reported expenditures. First, since these pilot surveys involved recruitment of 60 clusters per survey instead of the standard 30 [7], we divided the per diem costs of the surveys by half. We did not adjust transportation, supply, or other costs, as these were less clearly related to the number of clusters. Second, since the pilot surveys required more intensive training compared with the expected future training efforts for TT-only surveys, which may be integrated with TIS and TSS trainings, we used the mean training costs by survey from the TIS and TSS conducted in the same country. The standard trainings for TT-only surveys should require a similar level of resources as TIS and TSS trainings. We did not adjust the costs of the standard TT-only surveys conducted in Nepal. We then compared the summary costs of TT-only surveys with those of TIS and TSS. In addition, since we expected costs to vary by country, we compared the cost per survey of TT-only surveys with the median cost per survey of TIS and TSS within the same country.

## Results

### TIS and TSS

**Fig 1:**
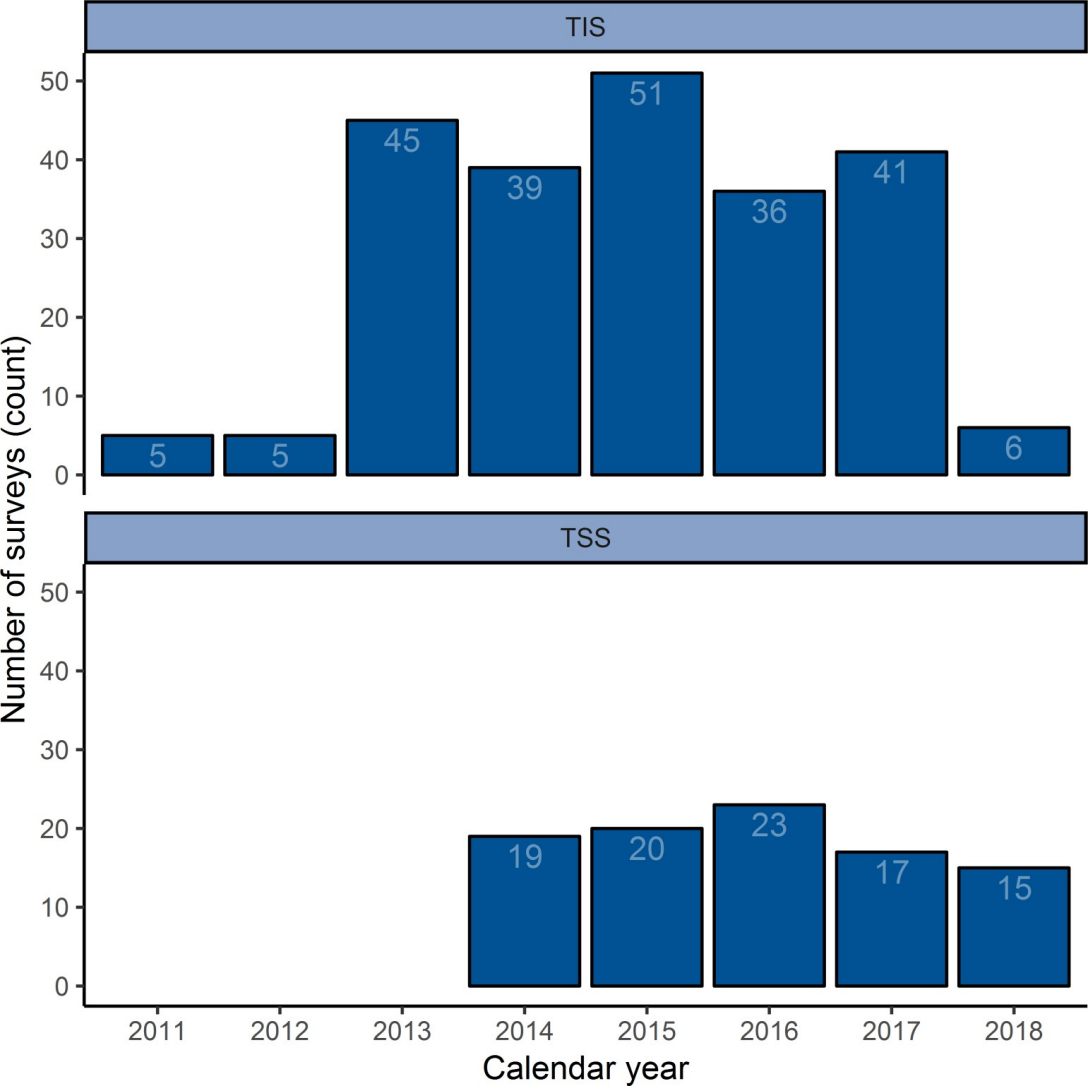
Number of TIS and TSS conducted per year. Each bar shows the number of each type of survey conducted in each year. As each EU must conduct TIS before TSS, TSS began being conducted in 2014, 3 years after the first TIS in our study.

**Fig 2:**
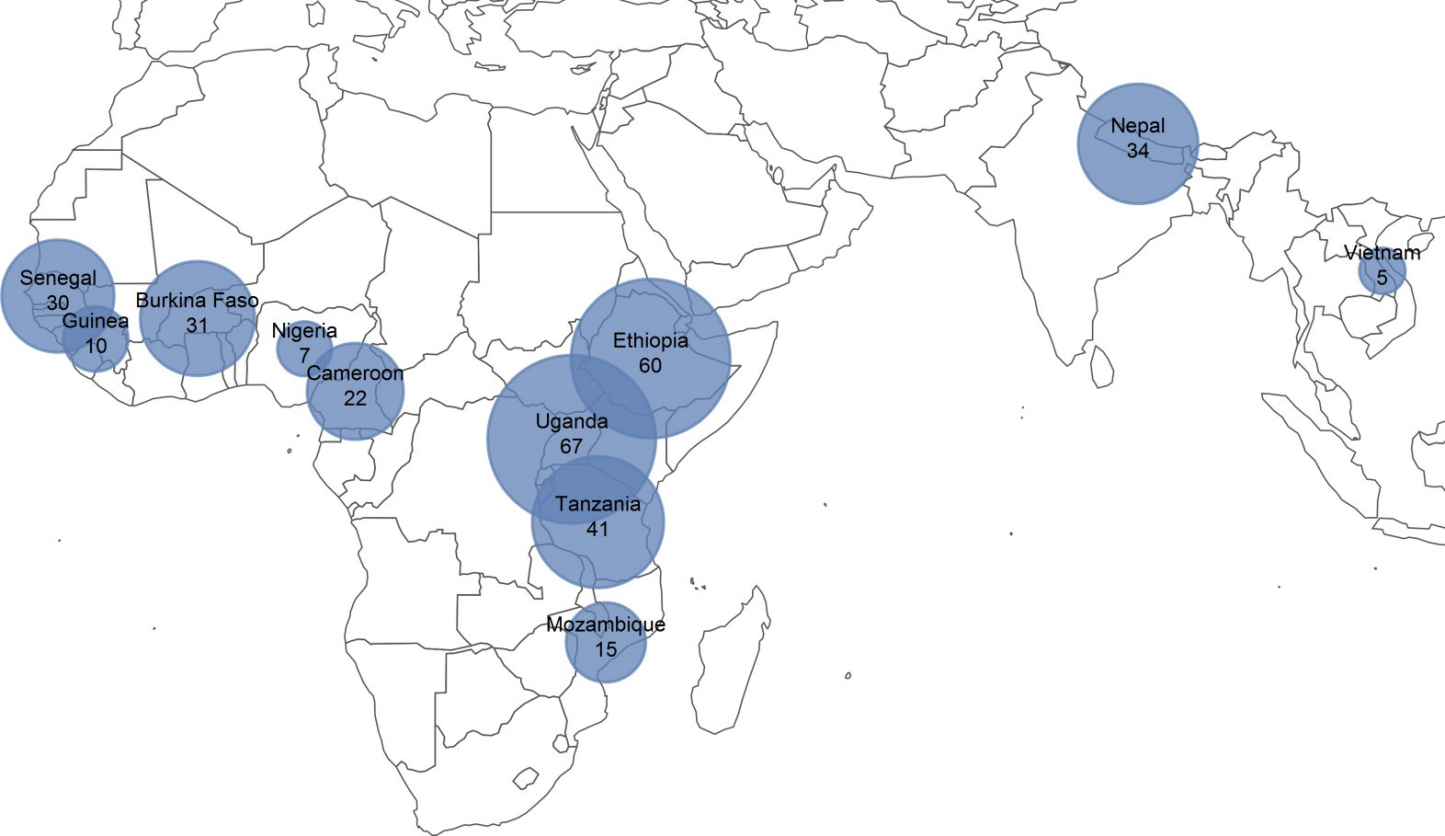
Number of TIS and TSS conducted in each country. The size of the bubble centered on each country represents the number of TIS and TSS included in the study from each country. Figure created by authors using R package rworldmap [17].

We gathered data from 228 TIS and 94 TSS, or 322 surveys in total. Surveys were conducted between 2011 and 2018, with the first TSS starting in 2014 (Fig 1). They took place in 11 countries: Burkina Faso, Cameroon, Ethiopia, Guinea, Mozambique, Nepal, Nigeria, Senegal, Tanzania, Uganda, and Vietnam (Fig 2). 250 surveys were funded by reimbursement of expenditures and 72 by FOGs.

**Fig 3:**
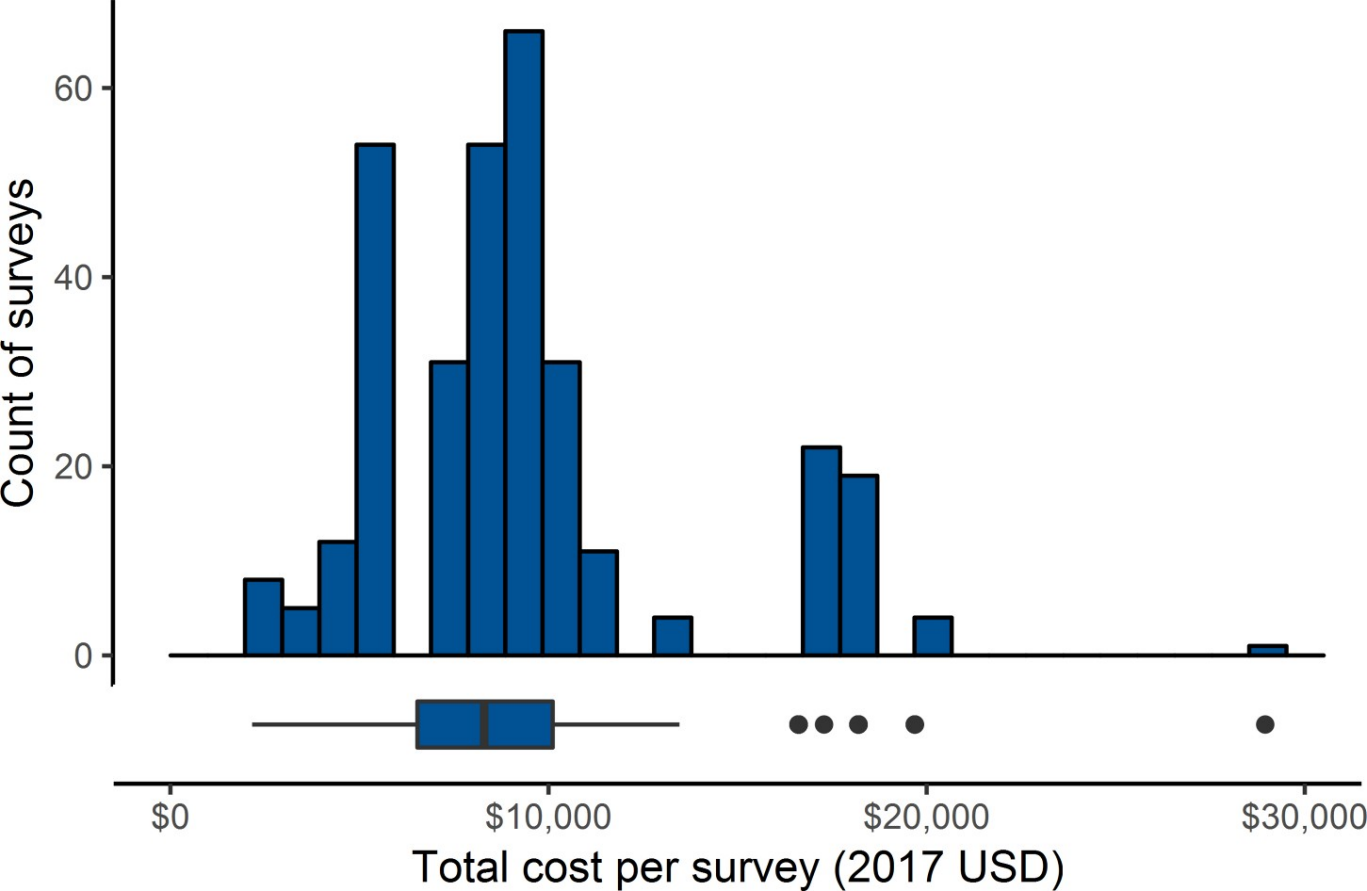
TIS and TSS cost distributions. The histogram displays the cost per TIS and TSS with bins $1,000 (2017 USD) wide. The boxplot, which shares its x axis with the histogram, further summarizes the data. The bar in the middle of the boxplot shows the median, and the edges of the box represent the interquartile range (IQR), or the 25^th^ and 75^th^ percentiles. The whiskers display the spread of the rest of the data, except for the outliers, defined as observations that fall more than 1.5 times the IQR away from the median, which appear as dots.

Fig 3 summarizes the overall costs per survey. (A more detailed breakdown of the data by country, survey type, year, cost category, and activity appears in S1 Data; the Q-Q plot of the distribution is shown in S1 Fig) As is common with cost data, a few high outliers created a skewed distribution of costs, which makes the median and interquartile range (IQR) better measures of their central tendency and spread than the mean and standard deviation. TIS and TSS cost a median of $8,298 (IQR: $6,532 –$10,111). Costs did not differ significantly by survey type (Mann-Whitney p = 0.68, 95% confidence interval (95% CI) for cost difference = -$620 –$788). Costs also did not differ significantly by the source of the data, FOG budgets versus expenditure records (Mann-Whitney p = 0.58, 95% CI = -$1,339 –$483).

Initially, in a simple linear regression, the cost per survey appeared to decline significantly by $407 per year of implementation in a country (95% CI = -$814 –-$46, adjusted R^2^ = 0.007). After controlling for country by adding each country as an independent fixed variable, however, the association between years of implementation and cost per survey was no longer significant (95% CI: -$222 –$607, model adjusted R^2^ = 0.69). In fact, differences between countries account for 69% of the variation in survey costs (adjusted R^2^ from a linear regression with each country as an independent dummy variable, bootstrapped 95% CI = 56%– 77%). In addition, the calendar year of implementation did not appear to be significantly associated with the cost per survey (95% CI: -$194 –$359, adjusted R^2^ = -0.002).

In a simple linear regression, the cost per survey declined by $98 per survey implemented in a single round (95% CI: -$125 –-$70, adjusted R^2^ = 0.06). After adding each country to the model as an independent fixed variable, this change shrank to an $83 reduction per survey implemented in a single round, but it remained statistically significant. (95% CI: -$108 –-$63, adjusted R^2^ = 0.71). See S2 Fig for histograms and Q-Q plots of these bootstrapped distributions.

The cost breakdown (Table 2) highlights the cost categories and activities that drive differing costs of TIS and TSS. By activity, survey fieldwork accounted for 80% of the total costs. Of those fieldwork costs, 58% went towards per diems, including lodging, and 38% went towards travel. Training accounted for the next largest proportion of costs by activity, at 13% of all costs. By cost category, “other” costs, mostly hall rentals and communications costs, accounted for 11% of costs.

**Table 2:** TIS and TSS costs by category and activity.

Activity / Category	Per diem	Travel	Supplies	Other	(all)
**Pre-meetings**	1%	0%	0%	0%	**2%**
**Training**	3%	2%	1%	7%	**13%**
**Survey fieldwork**	46%	30%	3%	1%	**80%**
**Survey design, data analysis, or report writing**	1%	0%	0%	0%	**2%**
**Post-meetings**	1%	1%	0%	0%	**2%**
**Other**	0%	0%	0%	2%	**2%**
**(all)**	**52%**	**33%**	**4%**	**11%**	**100%**

This table shows the breakdown of all costs by category and by activity, calculated as the proportion spent in each category and activity compared to all spending on TIS and TSS. Rows and columns may not sum to 100% due to rounding errors.

### TT-only surveys

Among the eight TT-only surveys conducted—three pilot surveys (one each in Cameroon, Tanzania, and Uganda) plus five routine surveys in Nepal—the median cost per survey was $9,707 (IQR $8,539 –$11,635), 119% of the median cost of a TIS or TSS. Compared with the median costs of TIS and TSS conducted in the same country, TT-only surveys cost a median of 106% as much (IQR: 94%– 136%). Given the small number of TT-only surveys in this study, we did not statistically evaluate the existence of a difference in costs between the costs of TIS and TSS versus the costs of TT-only surveys.

## Discussion

As in previous costing studies of trachoma prevalence surveys, costs per survey varied substantially by country and were driven primarily by the costs of per diems and travel for survey fieldwork [8–10]. Our estimated costs per survey [for TIS and TSS $8,298 (IQR: $6,532 –$10,111)] were substantially lower than those reported by Trotignon et al. for the Global Trachoma Mapping Project [median $15,839 (IQR: $10,773 –$19,915), 2015 USD; inflated to $16,316 (IQR $11,098 - $20,515), 2017 USD] [9]. They were higher than the Chen et al. costs [median $4,784 (IQR: $3,508 –$6,650), average of 2007–2009 USD; inflated to $5,498 (IQR $4,031 –$7,643), 2017 USD)]. The costs found in our study do align well with the costs found in a study which used very similar methods and inclusion criteria as ours to examine transmission assessment surveys for lymphatic filariasis, another cluster-based prevalence survey [median $9,540, 2012 USD; inflated to $10,298, 2017 USD; excludes rapid diagnostic tests] [18].

As noted in the discussion of the costing study conducted by Trotignon et al., the inclusion and exclusion criteria for costs can create substantial variation in the overall estimates [9]. A key difference in the inclusion/exclusion criteria of studies published to date was whether the data included international technical assistance—Trotignon et al. did, but Chen et al. and we did not. Furthermore, Trotignon et al. estimated the value of in-kind support from government partners in terms of transportation. As we estimated the financial costs to the national program itself, we excluded this support from our costing; given the high proportion of costs dedicated to transportation in both studies, this difference explains much of the discrepancy in expected survey costs. Other cost differences between the studies could derive from differences in the survey settings based on between-country and within-country features. For example, although Chen et al. reported a lower median cost per survey than we found, even within countries, the highest costs per survey found in our studies were similar ($28,954 in our study, $29,203 in Chen et al., both in 2017 USD). These highest costs in Chen et al. were associated with physical and logistical barriers that drove up transportation and personnel costs, even when compared with other districts in the same country [8].

Given the need to conduct surveys at a high level of quality [19] and according to standardized protocol templates [6,7], many of the component costs for TIS, TSS, and TT-only surveys are inflexible. Within countries, we found that the costs per survey did not decline over years of implementation or over calendar years; that is, even implementation experience did not help national programs find efficiencies that significantly affected their total costs. We did, however, find evidence of small economies of scale within a given country and round of survey implementation. Costs such as training costs, which do not increase linearly with the number of surveys as the per diems and supplies required for survey field work do, drive this difference. Inherent features of the area to be surveyed can keep costs fixed: external conditions of geography, population density, and seasonal effects affect the operations and therefore the costs of the survey [9]. The prevailing rates of per diems, vehicle hire, and fuel, in particular, dictate the costs of per diems and travel, which together account for 85% of the cost of the surveys in this study. In our study, countries whose surveys required a higher per diem for surveyors or greater expenditures on fuel due to higher prices or greater distances reported higher overall survey costs. Small efficiency gains found within a country over time might be offset by increases in the unit costs of these inputs or in resource needs for harder-to-reach survey locations [20]. In addition, the standard implementation methods used in these surveys already account for years of lessons learned in diverse international settings, which further decreases the likelihood of massive improvements in efficiency over time, though without rendering such improvements impossible.

TT-only surveys appear to cost about the same as or slightly more than TIS and TSS in the same country. This similarity makes sense due to the close resemblance of their methods. The slightly higher costs per TT-only survey could be attributed to the fact that TIS and TSS may include between 20–30 clusters while TT-only surveys almost always require 30 clusters [6,7]. We do note, however, that the small number of TT-only surveys analyzed in this paper limits the generalizability of these findings. We included these surveys in this paper to provide some insight into the potential costs of TT-only surveys, but a more robust future study of TT-only survey costs, once more are performed, would be useful.

The costs of surveys funded through FOGs and the costs of surveys funded directly did not differ significantly. As countries move toward self-reliance in their neglected tropical disease and other development programs, donor organizations have turned to FOGs and similar milestone-based grants (e.g., fixed amount awards) as a means of improving the management capacity of local health systems. For example, a recent evaluation of USAID’s Neglected Tropical Disease Program found that the use of FOGs improved communication and collaboration between the local governments that signed the grants and the local health service units that bore responsibility for achieving the milestones, which in turn increased the local governments’ interest and involvement in the health concerns of their communities [21]. Given this interest in FOGs, it is useful to note that they do not appear to change the cost of survey activities compared to methods that rely on tracking actual expenditures. Brady et al. found a similar lack of evidence of difference in costs by funding mechanism in their analysis of transmission assessment surveys for lymphatic filariasis [18].

The data and methods of this analysis, though in line with standards for costing analyses of this sort, do have several limitations. First and foremost, our choice of perspective of incremental financial costs to the national program excludes several key cost elements, including remuneration of full-time staff—except when they received per diems for survey activities—and the value of in-kind contributions, including vehicles and training space. It also excludes international training-of-trainer events. Although these events are a key piece of the quality assurance process for trachoma prevalence surveys [19], they are budgeted by a different mechanism and may support many more trachoma elimination projects than those accounted for here. Second, we calculated costs per survey by dividing the total costs of each type of survey in a country each year by the number of each type of survey conducted. This lack of EU-level costing obscures differences within a country, which prevents the analysis of factors such as distance between clusters that might drive differences in survey costs beyond country-level effects. Third, we assumed that all materials purchased for the surveys have a useful life of a single year and therefore did not amortize costs over multiple years, which could make early surveys within a country appear more expensive than later surveys even if they used the same amount of resources. In our analysis, however, we found no statistically significant change in within-country survey costs over time, which suggests that this assumption was appropriate. Finally, although this paper evaluates data for all surveys available to the authors, it is possible that our findings in this paper might not apply equally to surveys in other environments. For example, as all of the surveys examined used Tropical Data for data capture, this study cannot estimate the expected costs of a paper-based survey method or of surveys not using the standardized Tropical Data methodology. WHO, however, does now recommend the use of electronic data capture for trachoma prevalence surveys [6]. We hope that our analyses provide sufficient context for national programs to adapt the findings for use elsewhere.

Although these objective, standardized, population-based surveys require substantial investment, they provide national programs with the data necessary to plan their trachoma intervention activities. Furthermore, they improve confidence that the monitoring of progress toward WHO elimination prevalence thresholds is taking place with as much care as baseline mapping was performed [19,22]. Costing studies like this one therefore provide crucial planning data as countries advance toward the elimination of trachoma as a public health problem.

## Supporting information

S1 Data Detailed data. This workbook presents the detailed expenditure information by country, fiscal year, survey type, category, and activity. Due to the structures of the expenditure records used for this study, the years of the surveys are presented according to the United States government fiscal year, which begins in the October of the previous calendar year and ends in September of the given calendar year. For example, fiscal year 2017 ran from October 1, 2016 through September 30, 2017. The TT-only costs in the TT-only tab are presented with the adjustments described in the methods section.
(XLSX)

S1 Fig Q-Q plot of costs per survey vs. normal distribution. This Q-Q plot compares the actual distribution of costs per survey (the points) with the normal distribution (the lines). The shape of the points illustrates a right-skewed distribution of costs.
(TIF)

S2 Fig Histograms and Q-Q plots of bootstrapped distributions. These histograms show the bootstrapped distributions, with bootstrapped 95% confidence intervals and observed point estimates, of the listed variables. The Q-Q plot to the right of each histogram compares the bootstrapped distributions with the normal distribution. All distributions show deviations from the expected normal distribution. The bias-corrected and accelerated confidence intervals should correct for the deviations from the normal distribution seen in these plots.
(TIF)
