# Supplementary figures and images for: The costs of monitoring trachoma elimination: Impact, surveillance, and trachomatous trichiasis (TT)-only surveys
Source: PLoS Negl Trop Dis. 2019 Sep 5;13(9):e0007605. doi: 10.1371/journal.pntd.0007605 (PMC6728015; doi:10.1371/journal.pntd.0007605)

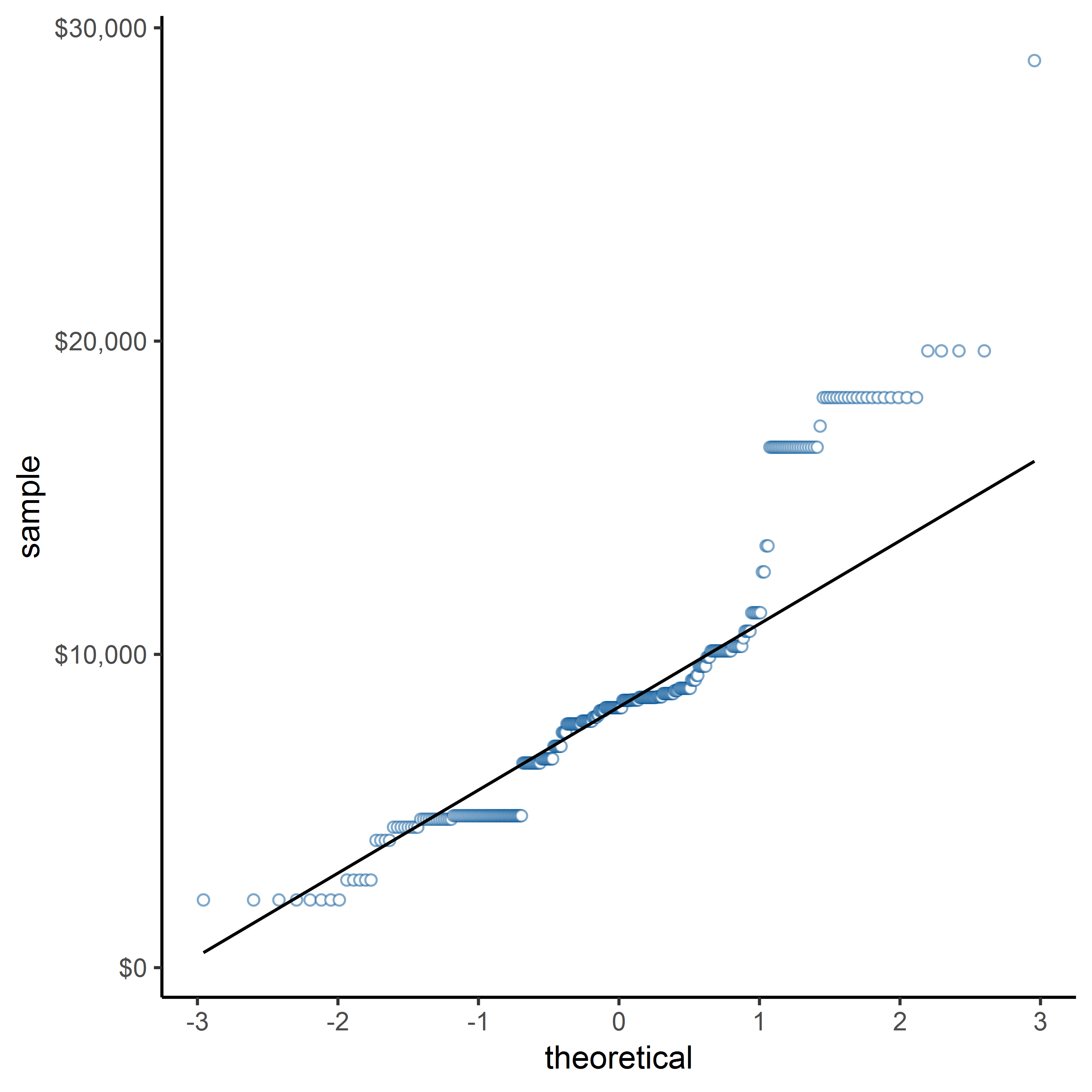

Supplement: S1 Fig — This Q-Q plot compares the actual distribution of costs per survey (the points) with the normal distribution (the lines). The shape of the points illustrates a right-skewed distribution of costs. (TIF) [file pntd.0007605.s002.tif]

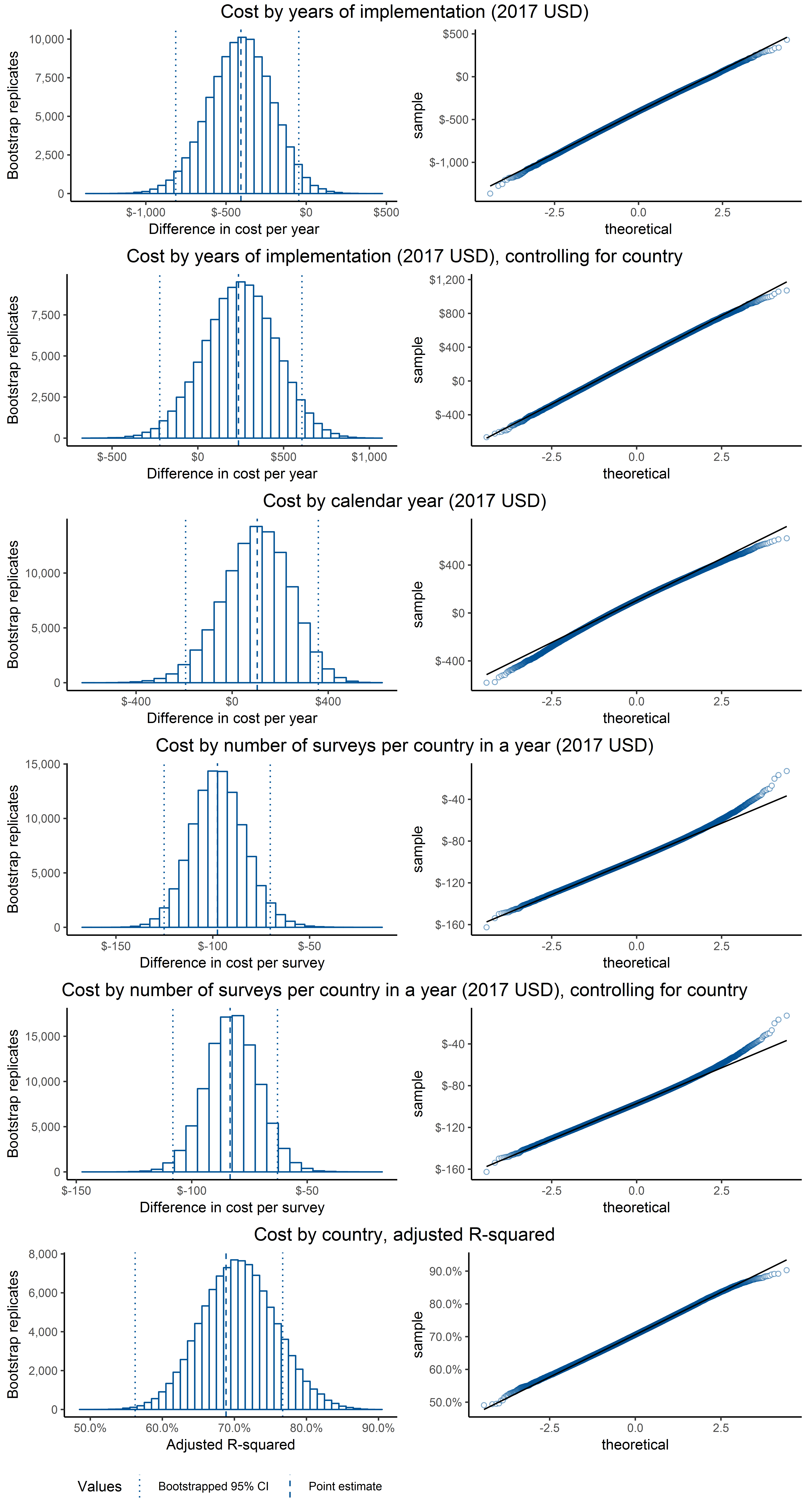

Supplement: S2 Fig — These histograms show the bootstrapped distributions, with bootstrapped 95% confidence intervals and observed point estimates, of the listed variables. The Q-Q plot to the right of each histogram compares the bootstrapped distributions with the normal distribution. All distributions show deviations from the expected normal distribution. The bias-corrected and accelerated confidence intervals should correct for the deviations from the normal distribution seen in these plots. (TIF) [file pntd.0007605.s003.tif]
